# How genome ‘escape’ from mechanical stress-induced damage

**DOI:** 10.1038/s41392-020-00227-2

**Published:** 2020-06-30

**Authors:** Xiaoyun Dai

**Affiliations:** 1 Department of Genetics, Yale University School of Medicine, New Haven, CT USA; 2 System Biology Institute, Yale University, West Haven, CT USA; 3 Center for Cancer Systems Biology, Yale University, West Haven, CT USA

**Keywords:** Cell biology, Molecular biology

**Fig. 1:**
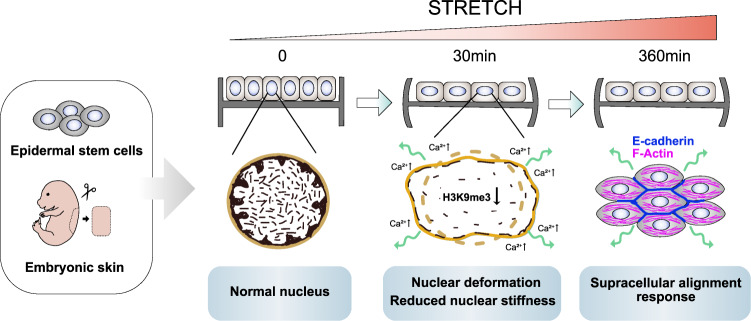
Genome is protected against mechanical stress-induced damage by rapid chromatin mechanoresponse and slower supracellular alignment response. The human epidermal/progenitor cells (EPCs) and E15.5 mouse embryonic skin was used by investigators

**A recent study by Dr. Wickström et al. published in*Cell*reveals a new mechanism for how genome is protected from mechanical stress-induced damage. The study demonstrates that cells show rapid chromatin mechanoresponse by loss of H3K9me3-marked heterochromatin and tissue-level reorganization to prevent long-term stretch-induced damage (Fig**. 1**).**

Homeostasis of epithelial tissues are essential for maintenance of functional integrity under force-driven deformations.^1^ Despite tissue integrity can be impaired by extreme mechanical stress within tissues,^2,3^ epithelial sheets have the ability to sustain large deformation and mechanical stretch by changing cell shape or cell contacts during morphogenesis without signs of damage.^4^ However, it is unclear how mechanical energy is redistributed within the nucleus, and how genome is protected in respond to mechanical stress. Michele M. Nava at Helsinki Institute of Life Science and his colleagues led a new study to investigate the underlying mechanisms of genome mechanoprotection.^5^

To interrogate such mechanisms and properties, the investigators exposed human epidermis stem/progenitor cell (EPC) monolayers to distinct amplitudes of mechanical stretch. With the low-amplitude stretch, the authors observed that filamentous actin (F-actin) specially around nucleus reoriented to the direction of stretch in a time-dependent manner. Mechanical stretch deformed the nucleus with increased nuclear envelope wrinkling, but it had no substantial effect on nuclear volume. To characterize the mechanisms of the mechanic stress-induced changes in nuclear envelope tension, the phosphoproteomic analysis was performed after cells exposed to different amplitudes of stretch. The results implied that low-amplitude cyclic stretch-induced a downregulation of histone H3 lysine 9 trimethylation (H3K9me3), and this stretch-induced heterochromatin response was further validated by H3K9me3 immunofluorescence staining. Moreover, the authors also used the chromatin immunoprecipitation sequencing (ChIP-seq) to profile genome-wide changes in H3K9me3 from stretched cells. The team observed that the downregulated H3K9me3 occupancy was observed particularly at non-coding regions and few occurred at protein-coding regions. Due to the motif enrichment of calcium (Ca^2+^)/calmodulin-dependent protein kinase II consensus sites from the phosphoproteomics data, the authors hypothesized that the nuclear deformation possible triggered Ca^2+^ signaling pathway. By using live imaging of intracellular Ca^2+^, the authors identified that deformation of the nuclear envelope increased Ca^2+^ release from the endoplasmic reticulum (ER), and which was mediated by Piezo ion channel to reduce nuclear membrane tension and lamina-associated H3K9me3 heterochromatin.

After insistent exposure EPCs to high-amplitude stretch, the authors found that the adherent junctions were reoriented into a 45° angle to the stretch axis, and the decreased in H3K9me3 at lower-amplitude stretch was reversed after continuous stretch. According to phosphoproteomics data analysis, another large cluster showed that phosphosite at actin and adhesion regulators such as myosin-9, cofilin, and paxillin remained downregulated at 360 min stretch. This was relative to the dynamics of supracellular alignment. These data indicated that EPC monolayer alignment perpendicular to stretch was not dependent on the nuclear deformation pathway, and force was redistributed by cell-cell contacts to prevent mechanical energy transmission to the nucleus. Next, the authors established an ex vivo system by using the E15.5 embryonic intact skin to test out whether the changes in chromatin architecture and supracellular monolayer alignment observed in vitro are also relevant in ex vivo. As expected, a transient reduction of heterochromatin was observed in both the basal stem cell and suprabasal layers under effect of cyclic stretch, after which the reduced expression of H3K9me3 was reversed by supracellular alignment.

Overall, the significance of the findings is that the authors identified two distinct mechanisms to keep genome integrity in response to different amplitudes of stretch. First, cells show rapid chromatin mechanoresponse via Piezo1 ion channel-mediated Ca^2+^ release from the ER, which is driven by downregulation of H3K9me3-marked heterochromatin. Second, if the mechanical stress persists, the long-term stretch results in tissue-level reorganization to spread out mechanical energy to prevent force transfer to the nucleus (Fig. 1). These unconventional findings are likely to have broad significance in different type of cells and model systems.
